# Development of an intravaginal ring delivering simultaneously anastrozole and levonorgestrel: a pharmacokinetic perspective

**DOI:** 10.1080/10717544.2019.1622609

**Published:** 2019-06-07

**Authors:** Rüdiger Nave

**Affiliations:** Translational Medicine, Bayer AG, Berlin, Germany

**Keywords:** Clinical pharmacokinetics, women's health, gynecology, drug delivery

## Abstract

Intravaginal rings (IVRs) are an option for continuous administration of drugs in women. As an attractive approach for the treatment of endometriosis-associated pelvic pain, IVRs delivering a combination of the aromatase inhibitor anastrozole (ATZ) and the progestin levonorgestrel (LNG) have been developed. This article describes the developmental pharmacokinetic (PK) aspects covering the characterization of *in vitro* release, preclinical IVR PK investigations in monkeys, and clinical PK considerations. An IVR for ATZ has been developed and investigated in healthy menstruating female cynomolgus monkeys showing effective *in vivo* release. PK data from the size-adapted IVR used in these animals can be translated into a human context as confirmed in human studies where predefined exposure levels of ATZ were reached. As ATZ may cause harm to the fetus, use of effective contraception has to be assured in women of childbearing potential. Therefore, the IVR delivers a low dose of LNG as a contraceptive. Although the daily dose differed strongly between both drugs (20 µg LNG/d to >1 mg ATZ/d), simultaneous delivery of ATZ and LNG *in vitro* and *in vivo* was observed with a high correlation between the *in vitro* release and PK profiles. The PK characteristics successfully guided the design of clinical studies investigating the drug–drug interaction (DDI) potential. No relevant DDI between both the investigated or other vaginally administered drugs were identified.

## Introduction

Endometriosis is a chronic inflammatory disease affecting up to 10% of women of reproductive age (Kennedy et al., 2005; Dunselman et al., 2014). There is a high unmet need for effective medical therapies with favorable safety profiles for the long-term treatment of endometriosis. After discovery of aromatase overexpression in endometriotic lesions, new treatment concepts have emerged that aim to reduce local estrogen production in endometriotic lesions through means such as utilizing aromatase inhibitors (Bulun et al., 2000). However, the aromatase inhibitor anastrozole (ATZ) may cause harm to the fetus as studies in animals have shown reproductive toxicity (Arimidex, 2017). Therefore, effective contraception has to be assured while using ATZ in women of childbearing potential.

We followed this concept to develop a fixed combination of an aromatase inhibitor with a contraceptive. The aromatase inhibitor ATZ and the progestin levonorgestrel (LNG) were chosen. ATZ is a marketed drug with well-documented pharmacological and safety profiles for oral administration (Plourde et al., 1994; Amsterdam et al., 2005; Arimidex, 2017). In an observational clinical study, daily oral administrations of 1 mg ATZ resulted in pain relief and lesion size reduction (Amsterdam et al., 2005). However, ATZ is only approved in postmenopausal breast cancer patients. LNG is a well-characterized contraceptive delivered by different routes of administration (e.g. as low-dose progestin-only LNG pill or LNG implant) (Bayer Norgeston, 2018; Bayer Jadelle, 2002).

We considered intravaginal administration, which has been shown as a feasible route for systemic delivery for various pharmaceutically active agents, such as steroids (Chien, 1992; Mishell et al., 1970; Hussain and Ahsan, 2005; Brache and Faundes, 2010). This route of administration can be advantageous as drugs absorbed from the vagina do not undergo first-pass metabolism due to blood from the vagina reaching systemic circulation via the internal iliac veins (Chien, 1992; Richardson et al., 1992; Hussain and Ahsan, 2005). This route is also used to provide sustained and controlled drug release, most often for contraceptive steroid hormones and hormone replacement therapy (Ballagh, 2001; Brache and Faundes, 2010). The first contraceptive trials using vaginal rings were conducted in the 1980s (Kerns and Darney, 2011). Nowadays, commercial intravaginal rings (IVRs) are available for contraceptive use including the NuvaRing^®^ that releases etonogestrel and ethinyl estradiol (Timmer and Mulders, 2000; Mulders and Dieben, 2001; van Laarhoven et al., 2002; Trussell, 2011). IVRs delivering LNG are not currently commercially available.

For the development of a new combination of drugs, the potential interaction between both drugs must be investigated while drug–drug interaction (DDI) with other drugs, in particular with regard to metabolism, should be known from the label of the marketed drugs. It is worthwhile to mention that ATZ inhibits CYP3A4 *in vitro* and that LNG is metabolized by CYP3A4 (Arimidex, 2017; Bayer Norgeston, 2018). Moreover, knowledge of potential effects of other intravaginally administered drugs on the pharmacokinetics (PK) of LNG and ATZ are important and, in this respect, even co-usage of tampons might be considered.

The objective of this article is to provide an overview on the development strategy for an IVR delivering ATZ and LNG simultaneously. With regards to data on the *in vitro* release rates, size-adapted IVRs were investigated in female cynomolgus monkeys with the resulting data then extrapolated for application in humans. In particular, PK topics to be addressed in the IVR development are discussed. PK properties related to this route of administration have shaped the design of clinical studies investigating the DDI potential.

## Materials and methods

### Intravaginal rings

**Figure 1.**
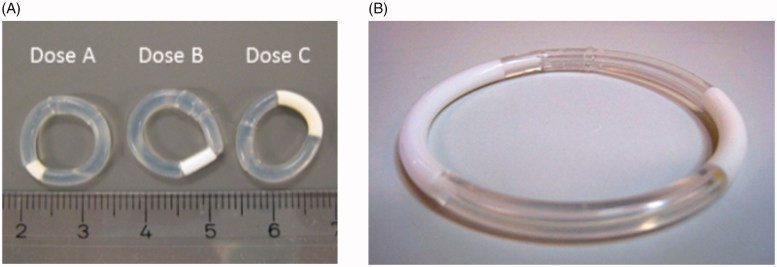
Intervaginal rings (IVR): (A) Monkey IVRs releasing ATZ; (B) Human IVRs releasing ATZ and LNG (54 mm diameter).

The IVRs were made of silicone elastomer and the drug core was covered on the outer surface by a single continuous transparent elastomeric membrane for controlled drug release. In terms of structural dimensions, the outer diameters were 14 and 54 mm for the IVRs used in female cynomolgus monkeys and humans, respectively (Rotgeri et al., 2015; Schultze-Mosgau et al., 2016). The different dose administrations were achieved by varying the length of drug segments responsible for releasing different doses of ATZ (Figure 1). IVRs releasing a combination of ATZ and LNG were developed for human use only. The intended wearing period is 28 days.

The IVR used in the phase 2b study with ATZ/LNG 1050/40 µg/d reflects the highest ATZ dose feasible for the current IVR system, given the segment length of 25 mm needed for release of 40 µg/d LNG.

The IVRs contain excess drug to enable a steady drug release. For example, the average LNG content in IVRs releasing 40 µg/d in early clinical studies was 177 mg. New LNG formulations developed to have a similar *in vitro* release rate profile, but a significantly lower LNG content have been designed (Nave et al., 2018). One key difference includes the smaller thickness of the drug-containing layer in the revised formulation that maintaines an average LNG content of 10.6 mg while using the same membrane for controlled drug release. So far this revised formulation with reduced LNG content is only available for mono-LNG IVRs.

### *In vitro* release

The *in vitro* release rates of ATZ and LNG IVRs were tested in 1% 2-hydroxypropyl-beta-cyclodextrin-water solution using a water bath incubator at 37 °C. With the exception of weekends, sampling was performed daily for up to 28 or 42 days for human and monkey IVRs, respectively (Rotgeri et al., 2015; Schultze-Mosgau et al., 2016; Nave et al., 2018). The concentration of ATZ and LNG were analyzed by liquid chromatography with UV detection (HPLC-UV).

### Cynomolgus monkey study

Female cynomolgus monkeys (*Macaca fascicularis*) aged 5–6 years with a median body weight of 3.5 kg and who also had a regular menstruation cycle were included in the study (Rotgeri et al., 2015). The study (study code: A 0451/09) was approved by the German animal welfare authorities (LAGeSo, Berlin). ATZ was administered with IVRs at 3 different dose levels (nominal *in vitro* release rates: 10, 50, 250 µg/d) with 5 animals per group. IVRs were fixed with a suture loop within the vagina for one menstrual cycle (up to 42 days). Blood samples to assess PK were collected at least every third day.

### Target exposure consideration

From safety perspective, the highest ATZ dose for human use should result in systemic exposures similar or lower to oral 1 mg per day, which is the approved dose for treatment of breast cancer in women after menopause (Arimidex, 2017; Plourde et al., 1994). Due to the long half-life (*t*_1/2_) of ATZ (50 h), the drug accumulates in steady state following daily single dose administrations (Higa and al Khouri, 1998). Mean *C*_trough_ values in steady state of 38.4 µg/L in postmemopausal women with breath cancer have been reported for daily doses of 1 mg (Wiseman and Adkins, 1998). Data from cynomolgus monkeys provide an insight into the relation between *in vitro* and *in vivo* release of ATZ.

A further aim was to find a dose of LNG that results in exposure similar to that reported for approved low-dose oral LNG formulations that have well established contraceptive effects (Bayer Norgeston, 2018). For LNG, contraception with daily oral doses of 30 µg was established and the mean average concentration (*C*_av_) following repeated oral administration of this dose is about 0.34 µg/L (Bayer Study 15687, 2013; Reinecke et al., 2018). An average concentration for LNG delivered from IVRs slightly above this *C*_av_ assuming safe contraception was targeted. This is also in line with LNG plasma concentrations from LNG implants after a wearing period of 2 years (Bayer Jadelle, 2002).

### Clinical studies

Clinical studies have been conducted and an overview is provided in Table 1. For the IVR, the dose per day for ATZ and LNG reflected nominal doses based on an intended *in vitro* release rate of the drugs at the end of the 28 day wearing period.

**Table 1.** Overview of clinical studies including PK investigations.

Study^c^	Objective	Nominal daily doses	*N*	Duration	Reference
FiH 2011-005620-18	PK, PD, safety, and tolerability	ATZ/LNG 500/20 µg/d ATZ/LNG 1000/30 µg/d ATZ/LNG 1500/40 µg/d^a^	21 19 20	56 days 56 days 56 days	Schultze-Mosgau et al., 2016
DDI (vaginal) 2014-005167-32	PK effects on ATZ/LNG of intravaginal: – Miconazole, – Clindamycin, – Nonoxynol-9, – Tampons	ATZ/LNG 1050/40 µg/d	11 12 13 13	16 days 16 days 16 days 35 days	Nave et al., 2018
DDI (phase 2b) 2013-005090-53	PK effects of ATZ on the PK of LNG	ATZ/LNG 300/40 µg/d ATZ/LNG 600/40 µg/d ATZ/LNG 1050/40 µg/d LNG 40 µg/d	40 51 47 44	84 days 84 days 84 days 84 days	Nave et al., 2019
mono-LNG IVR^d^	PK of a revised LNG formulation	LNG 40 µg/d LNG 40 µg/d (revised formulation)	13^b^ 13^b^	28 days 28 days	Nave et al., 2018

a 2 IVRs ATZ/LNG 750/20 µg/d.

b Same subjects (change over).

c EudraCT number.

d EudraCT number not applicable as study was performed in Japan.

The first in human (FiH) study was a randomized, open-label, multicenter, phase 1 study with 3 parallel groups of healthy young women (Schultze-Mosgau et al., 2016). The treatment duration was 56 days (IVR change after 28 days) after an ovulatory pretreatment cycle. It should be noted that for the high dose group (ATZ/LNG 1500/40 µg/d) two IVRs of 750 µg/d ATZ and 20 µg/d LNG were used and reflect the highest dose tested in the program. Based on frequent blood samples, PK parameters for the entire treatment period could be assessed.

A phase 1 DDI study was performed to investigate the effect of an intravaginally administered antimycotic, antibiotic, and a spermicide plus the co-usage of tampons on the PK of LNG and ATZ (Nave et al., 2018). In this parallel group, randomized, open-label study, healthy premenopausal women received an IVR releasing 1050 μg/d ATZ and 40 μg/d LNG as the main treatment for at least 16 days. Co-usage of tampon or co-medications (400 mg miconazole or 100 mg clindamycin or 75 mg nonoxynol-9) were administered on 3 consecutive days during treatment with IVR. In addition, an extended wearing duration of 35 days without IVR replacement was implemented and the elimination of ATZ and LNG after IVR removal was investigated in a single group. The selected dose corresponds to the high dose used in phase 2b and blood samples were taken frequently.

Recently, a randomized, parallel-group, multi-center phase 2b study in women with symptomatic endometriosis has been performed investigating the PK effects of ATZ on LNG among other objectives (Nave et al., 2019). Patients were randomized to the treatment group with IVRs releasing LNG 40 µg/d alone or in combination with ATZ 300, 600, or 1050 µg/d for 12 weeks. PK blood samples were taken at predose and before IVR replacement or removal (days 28, 56, and 84). The primary PK parameter was the plasma concentrations of ATZ and LNG at the end of each IVR wearing period.

A randomized, open-label, single-center, fixed-sequence phase 1 study in Japan was performed to investigate the PK of two formulations of mono-LNG IVRs (Nave et al., 2018). Although both formulations released 40 µg of LNG daily, there was a significant difference in the total amount of drug contained within the two mono-LNG IVRs. The treatment duration was 56 days with an IVR change from one to the other formulation after 28 days. The primary PK parameter was the plasma concentration of LNG at defined time points under stable conditions.

### Bioanalytics

HPLC methods with tandem mass spectrometry (LC-MS/MS) were developed and validated to determine ATZ concentrations in plasma of cynomolgus monkeys and humans. The lower limit of quantification (LLOQ) was 0.15 µg/L for monkey plasma and 0.05 µg/L for human plasma in the first clinical study (Rotgeri et al., 2015; Schultze-Mosgau et al., 2016). An LLOQ of 0.10 µg/L was reported in subsequent research studies (Nave et al., 2018). In addition, plasma concentrations of LNG in human subjects were determined using validated LC-MS/MS methods with an LLOQ of 0.05 µg/L or 0.01 µg/L in later studies (Schultze-Mosgau et al., 2016; Nave et al., 2018).

### Pharmacokinetic analysis

In almost all studies, the primary PK variable was the average concentration of ATZ and LNG in plasma at defined time points or intervals after insertion of the IVR. Where appropriate, other PK parameters like *C*_max_, *t*_max_, and *t*_1/2_ were calculated. All PK variables were analyzed by descriptive statistical methods.

Furthermore, population PK modeling for ATZ and LNG were conducted via a nonlinear mixed-effects approach considering the *in vitro* release data and residual content data to describe the *in vivo* release from the IVRs (Reinecke et al., 2017).

## Results

### *In vitro* release

#### *In vitro* release of ATZ

The nominal release rates for the monkey IVRs are 10, 50, and 250 µg/d. The actual *in vitro* release rate decreased over time with a decline in released ATZ (day 1 vs days 24–28) from 26 to 12, 85 to 54, and 390 to 271 µg/d (Supplemental Material, Figure S1).

Considering the body weight of humans and the geometry of the human vagina, larger size-adjusted IVRs with higher release rates were developed for the human use. For the FiH study, IVRs releasing one of the three ATZ/LNG dose combinations (*in vitro* nominal daily drug release rates on day 28: ATZ/LNG 500/20 µg/d, ATZ/LNG 1000/30 µg/d or ATZ/LNG 750/20 µg/d [high dose with 2 IVRs: 1500/40 µg/d]) were characterized *in vitro* (Schultze-Mosgau et al., 2016). Similarly, the *in vitro* release rate decreased slightly over time resulting in actual release rates on day 28 of 519, 975, and 1586 µg/d (Supplemental Material, Figure S1).

Overall, similar *in vitro* release rates for ATZ were obtained for IVRs used in the phase 2b study. Average actual *in vitro* release rates on day 28 of 290, 596, and 941 µg/d were reported for IVRs with nominal release rates of 300, 600, and 1050 μg/d (Nave et al., 2019).

In summary, the actual *in vitro* release rates from the ATZ-containing rings in monkeys were found to be as low as 12 µg/d, while the highest actual release rate from a single human IVR was 975 µg/d. For all IVRs containing ATZ, *in vitro* release rates are characterized by a slow decrease for up to 28 days.

#### *In vitro* release of LNG

For LNG, stable release rates over a period of 28 days were observed for all tested dose strengths. The actual *in vitro* release rates were slightly above the nominal *in vitro* release rates of 20, 30, and 40 µg/d (Schultze-Mosgau et al., 2016; Nave et al., 2019). There is obviously no difference in the *in vitro* release between IVRs delivering 40 µg/d LNG with or without ATZ (Supplemental Material, Figure S2).

**Figure 2.**
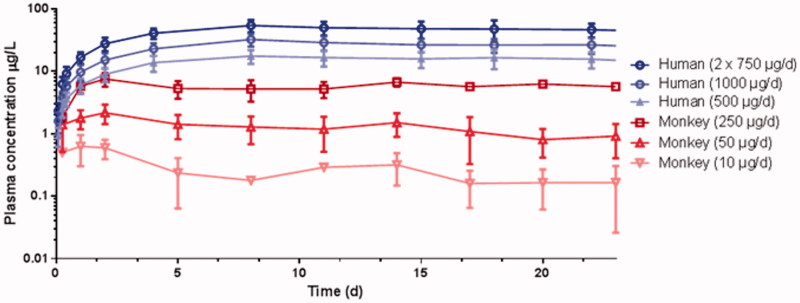
Mean (SD) ATZ plasma concentration time profiles after IVR insertion in female cynomolgus monkeys and young women.

Furthermore, the *in vitro* release rates for both formulations delivering LNG from the mono-LNG IVRs was investigated (Nave et al., 2018). Despite the fact that the drug content in the revised formulation was lowered by 94%, both formulations provided similar *in vitro* release rates. The relative difference for *in vitro* release rates over 28 days between both formulations was ∼1.2% (41.9 vs 41.4 µg/d).

### Pharmacokinetics

#### Pharmacokinetics of ATZ

Mean plasma concentrations of ATZ in monkey and human plasma increased steadily during the first few days after IVR insertion. The plasma concentrations increased in an approximately dose proportional manner between the tested doses. The concentration time profiles of ATZ in both species are very similar for this route of administration (Figure 2; Rotgeri et al., 2015; Schultze-Mosgau et al., 2016). The PK parameters are summarized in Table 2.

**Table 2.** Mean average (C_av_) ATZ plasma concentrations following insertion of first IVR.

	Monkey			Human		
Nominal dose ATZ µg/d	10	50	250	500^a^	1000^b^	1500^c^
*C*_av_ (µg/L)	0.29 (42)	1.4 (40)	5.9 (15)	14.1 (28)	24.5 (21)	43.0 (31)
*C*_av_/D (1/L)	0.029	0.035	0.024	0.028	0.025	0.029

Note: Animal data are in mean (%CV) while human data are provided as geometric mean (%CV).

a ATZ/LNG 500/20 µg/d.

b ATZ/LNG 1000/30 µg/d.

c 2 IVRs ATZ/LNG 750/20 µg/d.

Interestingly, similar dose normalized ATZ concentrations in steady state were observed in both species. PK data from the size-adapted IVR used in cynomolgus monkeys can be translated into a human context. It was assumed that *in vivo* and *in vitro* release rates were correlated and that these data indicate a high correlation between the *in vitro* release and PK profiles for both species.

In monkeys, IVRs were not replaced to investigate longer wearing periods. The intended wearing period for patients is 28 days followed by IVR replacements every 28 days to allow for continuous treatment. Therefore, in the FiH study, PK of ATZ including replacement of IVRs was investigated. As expected, the exposure is quite stable with geometric mean *C*_av_ of 15.5, 27.4, and 49.1 µg/L for the second wearing period for the above mentioned IVRs (Schultze-Mosgau et al., 2016).

Based on these data, a population PK model for ATZ was developed (Reinecke et al., 2017). The PK of ATZ was best described by a two-compartment model with linear elimination in conjunction with a constant, time-dependent first-order release rate for IVR delivery of ATZ. The observed covariate effects of body size parameters such as body weight were weak and not included in the model. Release rates of ATZ for the phase 2b dose-finding study were selected based on model simulations. It was assumed that 4 μg/L in plasma denotes the minimally effective biological activity (Plourde et al., 1994). As a result, the lower 90% confidence interval of geometric mean concentration at the end of the wearing periods should be covered by the lowest release rate. Based on simulation scenarios, the lowest release rate of 300 μg/d was chosen as the predicted minimally effective dose. Selection of the highest release rate of ATZ was not limited by safety issues as there were no safety concerns at the highest administered dose level (1500 µg/d) in the FiH study. However, the highest release rate for ATZ was limited by the technical feasibility of using only 1 IVR of an acceptable size in combination with LNG. Therefore, 1050 μg/d was chosen as the maximum dose.

The PK of this high dose (ATZ/LNG 1050/40 µg/d) was characterized in a DDI study resulting in geometric mean concentrations of 24.9 µg/L at the end of the wearing period (Nave et al., 2018). Similar *C*_trough_ values were observed for this dose in the phase 2b study (Nave et al., 2019). These values indicate that the systemic exposure following ∼1 mg ATZ per day using an IVR is comparable to a daily oral dose of 1 mg (Wiseman and Adkins, 1998; Schwartzberg et al., 2017; Plummer et al., 2018).

Based on the PK profile of ATZ in steady state within the IVR wearing period, collection of only 1 blood sample within the treatment period (independent of collection time) would provide sufficient information with regard to the exposure. For the phase 2b study, this concept was used and PK samples were taken prior to IVR exchange. To reduce variability, the median value from 3 samples was calculated and referenced as *C*_ss_. One outcome of the PK investigation within this study was the confirmation of dose proportionality of ATZ exposure in the range of 300–1050 µg/d (Nave et al., 2019).

A summary of the PK results across studies is provided in Table 3 together with the corresponding LNG data described in the next section.

**Table 3.** Geometric mean (CV%) *C*_trough_ values of ATZ and LNG in plasma for each study and dose group.

Study	Nominal daily doses^b^	Specified wearing period	ATZ (µg/L)	LNG (µg/L)
FiH (Schultze-Mosgau et al., 2016)	ATZ/LNG 500/20 µg/d ATZ/LNG 1000/30 µg/d ATZ/LNG 1500/40 µg/d^a^	2^b^	12.5 (53.6) 21.8 (27.5) 40.5 (39.3)	0.184 (69.1) 0.210 (66.9) 0.340 (36.0)
DDI (vaginal) (Nave et al., 2018)	ATZ/LNG 1050/40 µg/d	1^b^	24.9 (57.3)	0.338 (46.0)
DDI (Nave et al., 2019) (phase 2b)	ATZ/LNG 300/40 µg/d ATZ/LNG 600/40 µg/d ATZ/LNG 1050/40 µg/d LNG 40 µg/d	1–3^b^ (median)	7.85 (36.0) 15.5 (26.1) 22.6 (58.2) –	0.331 (48.8) 0.339 (50.8) 0.309 (54.6) 0.329 (46.5)
mono-LNG IVR (Nave et al., 2018)	LNG 40 µg/d	2^b^	–	0.314 (38.2)

a 2 IVRs ATZ/LNG 750 µg/20 µg.

b At the end of the wearing period.

#### Pharmacokinetics of LNG

**Figure 3.**
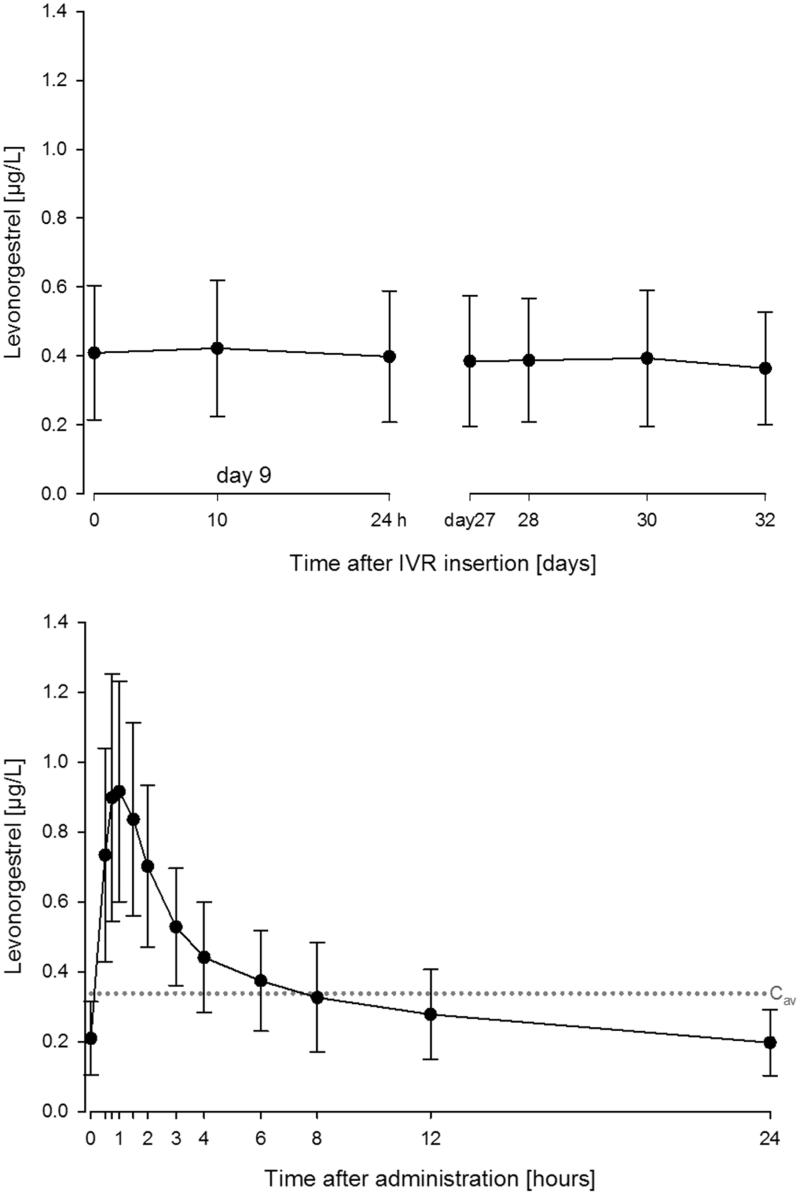
Mean (SD) concentration of LNG in plasma following insertion of an IVR (ATZ/LNG 1050/40 µg/d) on day 9 and after 4 weeks [top] and following repeated oral LNG doses (30 µg/d) for 4 weeks [bottom]. Dotted line: average concentration (*C*_av_) p.o. in steady state.

IVRs delivering the progestin LNG have not been tested in cynomolgus monkeys. In humans, plasma concentrations of LNG increased steadily following the first IVR insertion in healthy young women reaching *C*_max_ after ∼4 days (Schultze-Mosgau et al., 2016). Subsequently, daily and weekly LNG concentrations remained relatively constant. This is illustrated in Figure 3 with similar mean LNG concentrations on day 9 and day 28 (Nave et al., 2018).

Although significant, the lack of fluctuation in daily concentrations is expected for this route of administration and was observed in a study with 3 samples collected on the same day 9 days after first administration (Figure 3, top). In comparison to IVRs that maintain stable concentrations over time, differences have been observed in the concentration time profiles following oral administration. Microlut^®^/Norgeston^®^ are progestogen-only contraceptive pills containing 30 µg LNG and have a well-established PK profile (Bayer Norgeston, 2018; Reinecke et al., 2018). As shown in Figure 3 (bottom), a fourfold increase from *C*_trough_ to *C*_max_ following repeated doses of 30 µg LNG was observed and characterized as the daily peak–trough fluctuations (Bayer Study 15687, 2013; Reinecke et al., 2018). It should be noted that as the LNG concentration is <8 h above the *C*_av_ and therefore missing one tablet in steady state would further decline the plasma concentration of more than 50% within the next 24 h considering a *t*_1/2_ of 13–20 h.

One objective of the FiH study was to investigate the PK and pharmacodynamics (PD) of LNG and ATZ delivered from IVRs with a specific focus on investigating which LNG dose will reach the intended *C*_av_ in steady state (Schultze-Mosgau et al., 2016). Similar exposure levels of LNG were observed following replacement of the IVRs. For the high-dose group (ATZ/LNG 1500/40 µg/d), the geometric mean *C*_max_, *C*_av_, and *C*_trough_ were 0.436, 0.355, and 0.340 µg/L, respectively. This indicates that the target *C*_av_ was achieved by a nominal LNG dose of 40 µg/d. However, 2 IVRs have been used simultaneously with each delivering 20 µg/d of LNG. Using all available data (including PD parameters) from this study, PK/PD models were generated and a nominal dose of 40 μg/d LNG was selected for further development based on the resulting exposure–response analysis.

A summary of the PK results for LNG across studies is provided in Table 3.

#### Investigation of DDI

As described above for both drugs, the concentration at the end of the wearing period as determined by a single PK sample was deemed a sufficient indicator for exposures observed over the entire treatment duration. This information was critical in the phase 2b study investigating potential DDIs of ATZ on LNG by utilizing the median LNG plasma concentration at the end of the IVR wearing period as a primary PK parameter (Nave et al., 2019). All point estimates for the LNG PK parameter ratios between the mono- and combination IVR groups were close to 1 and the 90% confidence interval limits were in the 0.80–1.25 range which is typically used in bioequivalence studies. There was no evidence of drug-drug interaction of ATZ on LNG at any of the ATZ doses investigated. The PK data obtained in this phase 2b study made additional dedicated phase 1 studies on the DDI of both compounds superfluous.

Considering the route of administration, administration of other concomitant intravaginally applied medications (e.g. antimycotics and antibiotics for the treatment of vaginal infections) may be necessary for use in conjunction with the IVR. As a result, a study was performed to investigate the potential effect of an intravaginally administered antimycotic (miconazole), antibiotic (clindamycin), and a spermicide (nonoxynol-9) in conjunction with the co-usage of tampons on the PK of LNG and ATZ (Nave et al., 2018). Healthy premenopausal women received an IVR releasing ATZ/LNG 1050/40 μg/d as the main treatment. Co-medications were administered on 3 consecutive evenings during treatment with the IVR. The length of 3 days for antimycotic and antibiotic treatment was chosen as it reflects the current clinical standard of care for intended use. Consequently, a similar repeated dosing regimen was also implemented for the spermicide and tampon investigations. The primary PK parameter was the *C*_av_ of ATZ and LNG at defined time intervals (prior to, during and up to 7 days after the start of co-medication). As previously discussed, the *C*_av_ can be derived from a few blood samples. As a result, the individual reference exposure data are determined on the day before the tampon co-usage or the DDI investigations. At least 11 subjects per group completed the treatments. Very similar ATZ and LNG plasma levels were observed across all groups. The calculated ratios of *C*_av_ confirmed the absence of PK interactions, because all relevant point estimates and 90% confidence intervals were within the range of 0.80–1.25. In conclusion, no restrictions for the use of the IVR are needed. It should be mentioned that this study adhered to the standard recommendation for many vaginally administered drugs that suggest using medication in the evening prior to bedtime. Consequently, the design of the study differs from most other phase 1 studies in that the subjects stayed overnight during the DDI investigations while their normal daily activities remained largely unaffected. This study design was only possible due to the PK profile of the vaginal administered drugs preventing a need for frequent PK sample collection during the night.

#### Other PK investigations

For all investigations using IVRs, the time of IVR exchange or removal is a significant factor. Although the intended IVR wearing period is 28 days, in some instances, the IVR may have been worn longer. To account for this, the exposure on day 35 compared to day 28 was investigated in the reference group of the vaginal DDI study (Nave et al., 2018). The ATZ concentrations on days 28 and 35 were similar with an observed geometric mean concentration of 24.9 and 25.3 µg/L ATZ, respectively. The corresponding LNG plasma concentrations on days 28 and 35 were nearly identical as shown in Figure 3. Therefore, the impact of a slightly prolonged wearing period is low. Following IVR removal, the concentrations for both ATZ and LNG declined. The geometric mean *t*_1/2_ was 40.4 and 25.6 h for ATZ and LNG, respectively. These data are consistent with published data from other routes of administration. The *t*_1/2_ of ATZ and LNG are in the range of ∼50 and 13–20 h, respectively (Bayer Norgeston, 2018; Higa and al Khouri, 1998; Bayer Jadelle, 2002).

#### Potential revised formulation for LNG

For adjusting drug release from IVRs parameters like surface area of drug releasing segment, membrane material characteristics, membrane thickness, and drug concentration in drug segment are relevant. LNG monotherapy IVRs with reduced LNG content have been designed and produced (Nave et al., 2018). *In vitro* release rate testing confirmed the similarity of drug release of two IVR formulations both releasing 40 µg LNG per day *in vitro*. Moreover, a PK study was performed to investigate both formulations during the wearing period of 28 days. The primary PK metrics was the *C*_av_ of LNG in plasma at defined time points under stable conditions. Statistical evaluation resulted in point estimates for the LNG PK parameter ratios between the both formulations close to 1 indicating very similar delivery characteristics.

## Discussion

The *in vitro* testing of IVRs demonstated the desired behavior with regard to stable delivery of drugs over a prolonged time period. The delivered dose could be adjusted by varying the lengths of the drug segment within the IVR. Preclinical investigations are one of the first steps in developing IVRs for human use. Cynomolgus monkeys were selected as a relevant model species to investigate PD for predictive human translation as they allow for the use of an IVR and have hormone-dependent menstrual cycles similar to that of humans. Furthermore, these animal studies should provide further insight into the PK and relationship between *in vitro* and *in vivo* release data. The focus of these studies was the characterization of the aromatase inhibitor ATZ in cynomolgus monkeys. Size-adapted IVRs for the cynomolgus monkey, releasing ATZ at 3 dose levels in the range of 10–250 µg/d were designed and tested. The PK results with the stable concentration levels over the entire treatment period and the relationship between *in vitro* and *in vivo* release have been promising. The PD evaluations (quantification of estradiol as major surrogate for treatment efficacy) support the concept using ATZ (Rotgeri et al., 2015). In summary, a vaginal drug delivery system for ATZ has been developed and showed effective *in vivo* release in monkeys.

The next step in the transition of the IVR from animals to humans was to conduct an FiH study investigating PK and PD to confirm targeted systemic drug exposure. *In vitro* release guided the pharmaceutical formulation development. The clinical concept establishing an IVR releasing ATZ and LNG simultaneously was successfully reached. The primary aim was to identify well-tolerated doses of ATZ that are sufficient to block aromatase activity, and therefore, inhibit production of estrogens. The highest ATZ dose should result in exposures similar to the approved oral dose of 1 mg/d (Arimidex, 2017). A further aim of the study was to find a dose of LNG that results in exposure similar to that reported for approved low-dose oral LNG formulations that have well established contraceptive effects (Bayer Norgeston, 2018). In the phase 1 study, all IVRs releasing different dosing combinations of ATZ and LNG showed good safety and tolerability in healthy premenopausal women. Furthermore, a dose-dependent reduction in estradiol was found indicating the desired PD effect (Schultze-Mosgau et al., 2016). For both drugs, targeted exposure levels based on oral route of administration were reached. Overall, the relative bioavailability for both drugs pertaining to this route of administration is comparable to the oral routes based on *C*_av_ values. As indicated in Figure 3, a similar *C*_av_ for LNG is obtained for both routes of administration. In recent publications, geometric mean AUCs in steady state of 697 and 755 µg*h/L were reported for 1 mg ATZ in cancer patients (Plummer et al., 2018; Schwartzberg et al., 2017). The derived dose-normalized *C*_av_/D values were 0.029 and 0.031 1/L, which are considered comparable to the ATZ data for the IVR shown in Table 2. Studies investigating the bioavailability in comparison to an intravenous route of administration have not been performed.

PK/PD modeling guided the selection of doses for further investigation of this combination IVR. The LNG dose of 40 µg/d was selected as the mean concentration at the end of the wearing period and is slightly higher compared to *C*_av_ of orally LNG (30 µg/d) (Reinecke et al., 2017). Implementation of this LNG dose into the IVR restricted the ATZ dose to 1050 µg/d due to technical feasibility considering so far used materials and dimension of the IVRs. Consequently, in addition to the ATZ/LNG 1050/40 µg/d IVR, three further IVRs (ATZ/LNG 300/40 µg/d, ATZ/LNG 600/40 µg/d, and LNG 40 µg/d) were selected for the phase 2b study in patients based on PK/PD modeling. In addition, a contraceptive efficacy phase 3 study over one year using mono-LNG IVRs (40 µg/d) was planned and started (Bayer Study 16803, 2017). It was planned to collect PK samples in a subgroup of subjects after 6 and 12 months of treatment. However, due to an early study termination, the planned population PK and PK/PD analyses were not performed.

The PK characteristics guided the design and the objectives of the subsequent studies. As summarized in this article, the daily concentration levels of ATZ and LNG following the IVR reinsertion were stable over the entire 28 day wearing period. It should be noted that in contrast to LNG, the *C*_av_ for the entire period for ATZ is about 25% higher compared to the ATZ concentration measured at the end of the IVR wearing period (*C*_trough_). Nevertheless, plasma samples taken at the end of the wearing period are an indicator for the exposure of ATZ and LNG in steady state. Due to the absence of fluctuation, the daily plasma concentrations of both drugs remain relatively constant. As a result, a single PK sample reflects a reliable measure of the systemic exposure. In contrast to studies with oral drug administration, very few samples are required to allow for the investigation of PK objectives in IVR studies. In the phase 2b study, it was demonstrated by analyzing 3 samples per patient that there was no effect of ATZ on LNG and that ATZ exposure increased in dose-proportional manner. In a DDI phase 1 study, the effect of repeated intravaginally administered antimycotic, antibiotic, and spermicide plus the co-usage of tampons on the PK of LNG and ATZ was investigated. The calculated ratios of *C*_av_ confirmed the absence of PK interactions between ATZ/LNG released from IVR and the investigated drugs as well as tampons. Therefore, no restrictions for the use of the IVR are needed to continue the clinical program intended to treat endometriosis symptoms.

Considering potential DDIs, there are additional advantages with the use of IVRs compared to oral administration including the absence of potential food interactions and no relevant considerations are required to account for first-pass and gut-wall metabolism during drug development. Furthermore, self-administration of IVRs with longer dosing intervals is beneficial for compliance as it is deemed more convenient by some patients compared with the daily intake of a pill (Novak et al., 2003; Alexander et al., 2004). This is supported by the positive compliance rates observed in the phase 2b study.

A comparison of the available PK results across studies as shown in Table 3 confirms reproducible exposure for both drugs and a clear separation between dose groups. This is worthwhile to mention as the factor between some doses is relative small (e.g. between 30 and 40 µg/d for LNG and 300–500 µg/d for ATZ).

The formulation of the IVRs can be further improved by reducing the overall drug content. In the case of LNG, it was shown that it is possible to deliver similar amounts of LNG with an IVR *in vitro* and *in vivo*, even though the total drug content in the ring varied significantly. This underlines the importance of carefully designing the formulation in controlled release delivery systems to minimize the amount of excess drug content. Moreover, the role of PK studies was shown to be essential for adequately characterizing *in vivo* release behavior.

## Conclusions

A vaginal drug delivery system for ATZ has been successfully developed with effective *in vivo* release rates observed in monkeys. PK data from the size-adapted IVR used in cynomolgus monkeys can be translated into a human context and has been confirmed in human studies. As ATZ may cause harm to the fetus, effective use of contraceptives has to be assured in women of childbearing potential. Therefore, the IVR delivers a low dose of LNG as a contraceptive. Similarly to ATZ, predefined exposure levels were reached for LNG.

It was assumed that *in vivo* and *in vitro* release rates were correlated and these data indicate a high correlation between the *in vitro* release and PK profiles. The PK characteristics heavily influenced the design of clinical studies investigating the DDI potential. As a result of these studies, no relevant DDI between both drugs as well as DDI with other vaginally administered drugs were identified.

## Supplementary Material

Supplement.docx
