# Supplementary material for: Development of an intravaginal ring delivering simultaneously anastrozole and levonorgestrel: a pharmacokinetic perspective
Source: Drug Deliv. 2019 Jun 7;26(1):586–94. doi: 10.1080/10717544.2019.1622609 (PMC6567139; doi:10.1080/10717544.2019.1622609)
Supplement: Supplement.docx [file IDRD_A_1622609_SM8397.docx]

Supplement Material

In Vitro Release


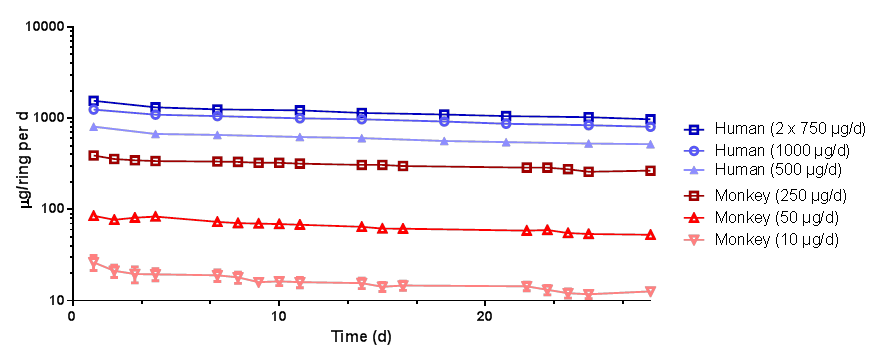


**Figure S1.** Mean (SD) *in vitro* release from ATZ IVRs


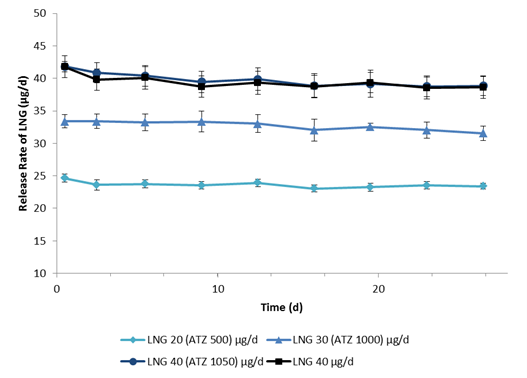


**Figure S2.** Mean (SD) *in vitro* release from LNG IVRs
